# Symbolic and non symbolic numerical representation in adults with and without developmental dyscalculia

**DOI:** 10.1186/1744-9081-8-55

**Published:** 2012-11-28

**Authors:** Tamar Furman, Orly Rubinsten

**Affiliations:** 1 Department of Learning Disabilities; Edmond J. Safra Brain Research Center for the Study of Learning Disabilities, University of Haifa, Haifa, Israel

## Abstract

**Background:**

The question whether Developmental Dyscalculia (DD; a deficit in the ability to process numerical information) is the result of deficiencies in the non symbolic numerical representation system (e.g., a group of dots) or in the symbolic numerical representation system (e.g., Arabic numerals) has been debated in scientific literature. It is accepted that the non symbolic system is divided into two different ranges, the subitizing range (i.e., quantities from 1-4) which is processed automatically and quickly, and the counting range (i.e., quantities larger than 4) which is an attention demanding procedure and is therefore processed serially and slowly. However, so far no study has tested the automaticity of symbolic and non symbolic representation in DD participants separately for the subitizing and the counting ranges.

**Methods:**

DD and control participants undergo a novel version of the Stroop task, i.e., the Enumeration Stroop. They were presented with a random series of between one and nine written digits, and were asked to name either the relevant written digit (in the symbolic task) or the relevant quantity of digits (in the non symbolic task) while ignoring the irrelevant aspect.

**Result:**

DD participants, unlike the control group, didn't show any congruency effect in the subitizing range of the non symbolic task.

**Conclusion:**

These findings suggest that DD may be impaired in the ability to process symbolic numerical information or in the ability to automatically associate the two systems (i.e., the symbolic vs. the non symbolic). Additionally DD have deficiencies in the non symbolic counting range.

## Introduction

Developmental dyscalculia (DD) is a specific disorder in numerical and mathematical abilities, with a neuro-anatomical source [1-3], for meta-analyses see: [4]. There is a continuous debate in scientific literature on the ability of people with DD to represent symbolic (e.g., Arabic numerals) and non-symbolic (e.g., a group of dots with different quantities) numerical information. In the current study we examined specifically whether DD adults are deficient in their ability to automatically process one or both of the systems of numerical representation. For this purpose, we used a novel version of the Stroop task [5] which we called enumeration Stroop. In the following introduction, we first describe these two numerical systems and then portray the rationale behind the method used to explore whether DD adults are deficient in one or both of these systems.

### Non symbolic numerical representations

Certain numerical skills, unlike reading skills, develop without formal teaching. These skills are commonly attributed to an analog, non symbolic, and approximate system [6,7]. Studies show that infants, and even animals, display several basic numerical skills such as counting, adding, and comparing [8]. Specifically, infants have been found to be not only capable of discerning small object sets (object tracking system), but also large sets [9,10] depending on visual-spatial processing capabilities [11].

DD is frequently attributed to a deficit in these basic, innate numerical processes, such as impaired understanding of the meaning of numbers or impaired quantity representation [1,12-14]. Some studies show that people suffering from DD encounter difficulties in automatically accessing numerical magnitudes [12,15,16]. However, developmental and brain imaging findings on DD and non symbolic number processing (e.g., comparing the numerosity of two groups of dot patterns) are inconclusive. Both group differences [3] and the absence of group differences [17] between children with and without DD were reported.

Enumeration develops during the first few years of life and has been suggested as essential for the proper development of numerical cognition. Discussions of enumeration distinguish between three processes: estimation, subitizing, and counting. In the current study we will examine subitizing and counting, which as opposed to estimation are both conscious and accurate [18].

Subitizing is an implicit cognitive ability to perceive small numbers [18,19]. That is, it is a fast, automatic, and accurate evaluation of a small set of objects (typically, 1 to 4 items; [19-21]). Very few studies have explored subitizing in DD participants. Koontz and Berch [21], for example, found that children with DD have a smaller subitizing range than the control group (see also [22] for similar observations). However, other studies did not manage to replicate these findings [23,24]. Moreover, subitizing has been shown to be trainable and can be enhanced in 7–9 year olds with mathematical deficiencies [25].

In contrast to subitizing, counting refers to larger sets of numbers and is a serial, symbolic, verbal, and effortful (i.e., requires working memory and attention resources) process [26,27]. In a typical enumeration task, the number of items in a set are named faster and more accurately in the subitizing range than in the counting range [19,20]. In the counting range, there is a linear increase of 200–400 ms per item [27]. This effect is not simply a numerical case of Weber's law, since it is evident only at the 1–10 range and not in the 10–20 range [28]; hence, subitizing and counting are processed differently. In support of this assumption, event related potential's (ERP) findings show that small quantities (1-4/3) are perceived as a single individual object, while large quantities are perceived as cardinal values [29]. There is a wide consensus regarding the existence of poor counting skills in the DD population [23,30,31]. For example, Geary, Bow-Thomas and Yao [32] conducted a series of counting tests with DD and control children. The results indicated a developmental delay in counting alongside incompetence in detecting counting errors in the DD group. In addition, Wilson and colleagues [25] did not manage to accelerate the counting rate in their training study. Therefore, counting, which requires attention and working memory resources, seems to be more deficient in the DD population than subitizing, which is an automatic process.

### Symbolic numerical representations

Symbolic numerical representations are distinct, accurate, and culturally-dependent (e.g., Arabic numerals such as “6” or number words such as “SIX” [33,34]). There is ample evidence that the non symbolic system is fundamental in the construction of symbolic numerical thinking [35,36].

DD is often attributed to a deficit in the ability to process symbolic representations; Rousselle and Noel [37] found that young DD children (age 7) were slower than age matched children only when comparing Arabic digits. They proposed that DD children may be slower than control children only in symbolic number processing. Mussolin, Martin and Schiltz [36] replicated these findings in adults with DD, and suggested that DDs have a "fuzzier" representation of symbolic number magnitude. Brain-imaging studies provide additional support for this hypothesis. Specifically, the parietal brain region has been shown to be less activated in young DD children (7–9 years old) than in control children when comparing symbolic numerical quantities (e.g., 3 vs., 8; [37,38]).

However, other findings support Dehaene’s [7] hypothesis suggesting a weak number sense in DD, meaning that both these systems, non symbolic (e.g., a group of dots) and symbolic (e.g., Arabic numerals) are impaired [39,40]. Recently, in a review paper, Noel and Rousselle [41] argued that the first deficit shown in DD emerges in symbolic numerical representations during the process of learning the symbolic numerical system. Deficiencies in non-symbolic numerical representations only appear later and are secondary to the first symbolic deficit.

### Ways of studying automaticity and attention in subitizing and counting

It has been suggested that numerical processing (symbolic and non symbolic) is automatic [12,42,43]. That is, this process begins immediately and even involuntarily upon seeing numbers. Psychologists use conflict situations in order to study automaticity. One such task is the Stroop task [5]. In this task, color-words are presented and participants are asked to name the color of the ink and ignore the meaning of the word. In many cases, participants cannot ignore the irrelevant dimension, which interferes with processing of the relevant one. Such a result is considered both a failure of selective attention and an indication of the automatic nature of the irrelevant dimension.

In the numerical Stroop paradigm (NSP) participants are presented with two Arabic digits and asked to compare either their numerical value (and ignore the irrelevant physical size) or their physical size (ignoring the irrelevant numerical value; e.g., congruent: 3 8; Incongruent: 3 8; [42]). In contrast to the classic Stroop, in the NSP the congruency effect (i.e., incongruent vs. congruent) is bidirectional, namely, irrelevant physical size can interfere with processing of the relevant numerical value, while irrelevant numerical value can also interfere with processing of the physical relevant size of the digit [12,42]. This is an indication that reading Arabic digits, as well as perceiving size, are both automatic processes. Recently, the NSP has been used to measure numerical automatic processes in the DD population. Dyscalculic participants, unlike healthy participants, fail to automatically process the irrelevant dimension [e.g., [13,44].

In the current study we will use a novel version of the NSP, **the enumeration Stroop**. In this task, participants are presented with a visual display containing a number of items (either in the subitizing range, 1 to 4 items, or in the counting range, 5 to 9 items). In the non symbolic task, participants are asked to report the number of items in the display while ignoring their identity. In the symbolic task participants are asked to report the identity of the presented items and ignore their quantity.

### Distance effect

Stroop tasks (such as the NSP) result not only in Stroop effects (i.e., congruent vs. incongruent) but also in a distance effect. The distance effect is an outcome of a decrease in reaction time (RT) and an increase in accuracy rate (acc) as a function of numerical difference between the written digits, or the quantities that are being compared [45]. For example, RT is typically shorter when one is asked to decide if 9 is larger than1, compared to a longer RT when comparing 9 to 8, and acc will be higher as well (in the case of 9 vs. 1). Numerical distance was found to affect children’s performance from an early age [46]. However, while some studies found that the slope of the distance effect (i.e., Y axis: RT or acc; X axis: the ascending distances) decreases with age [39,45], others have found no developmental change in the distance effect [46,47]. Additionally, a symbolic distance effect was found to be associated with mathematical proficiency [45].

Only few studies have attempted to examine the distance effect in DD participants. Mussolin et al. [48] examined young DD and control children (10–11 years old) on several tasks of numerical comparison. They found a distance effect regardless of the number format and an even stronger effect (a steeper slope) in DD children, as compared to a matched control group. A similar pattern was found in previous studies as well [3,49]. In contrast, other studies found no indication of a deviant pattern of distance effect, despite a significantly shorter RT [39]. To the best of our knowledge, there has been no study to date that has systematically examined the distance effect in adults with DD.

Henik and Tzelgov [42] found that manipulating the numerical distance in the NSP has an effect on performance, even if the distance manipulated was of the irrelevant dimension. Pavese and Umiltà [50,51] investigated the effect of symbolic distance between the two dimensions of a stimulus (i.e., relevant and irrelevant) in an enumeration Stroop resembling the one used in the current study (the task is presented in the section below). They found that the greater the numerical distance between the two numbers in the stimulus, the shorter the RT. Hence, the distance effect (i.e. the smaller the numerical distance, the longer the RT) is a potentially reversed force to the congruity effect (i.e. zero numerical distances, shortest RT) in the numerical Stroop tasks. This effect must evidently be taken into consideration when analyzing data from a Stroop-like task.

### The current research

**Figure 1:**
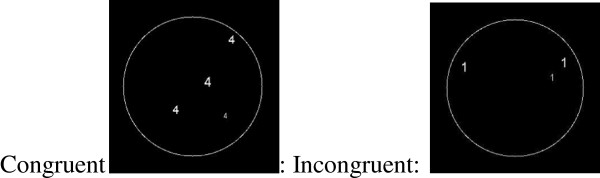
Examples of congruent stimuli and incongruent stimuli.

In the current study, we examined whether DD adults are deficient in one or both of the numerical representation systems, while analyzing levels of automaticity in the ability to count and subitize. For this purpose, a novel version of the NSP was used, which we call the **enumeration Stroop** (see Figure 1). In this task, participants are presented with a visual display containing a number of items (either in the subitizing range, 1 to 4 items, or in the counting range, 5 to 9 items). In the non symbolic task, participants are asked to report the number of items in the display while ignoring their identity. In the symbolic task participants are asked to report the identity of the presented items and ignore their quantity. The enumeration Stroop enables us to measure the automaticity of quantity (number of items) and numerical symbolic (Arabic numerals) perceptions, by comparing the different reaction times (RT) and accuracy (acc) of the congruent and incongruent trials (the congruency effect) in the symbolic task (i.e., measuring the automaticity of quantity processing) and in the non symbolic task (i.e., measuring the automaticity of symbolic processing).

The objective of the study is to examine whether there are significant differences between students with DD and typically developing students, in the novel enumeration Stroop task, as measured by reaction time and accuracy.

We predicted that **(1) In the non symbolic task** - incongruent, where the quantity (relevant dimension) is in the subitizing range in both the congruent and incongruent trials, we expected that the DD group will show a smaller congruency effect (incongruent vs. congruent) than the control group. We predicted this smaller effect since DDs show a deficiency in the symbolic system [37] and their perception of small quantities (i.e., subitizing) is intact [23,24]. Hence, we assumed that the irrelevant "weaker" dimension (the symbol) would not have an effect on the relevant "strong" one (the subitizing range). Where quantity (relevant dimension) is in the counting range in both the incongruent and congruent dimension, we expected to see the same effect in the two groups. We expected a similar effect in both groups because DDs are considered to be deficient in both the symbolic system [37] as well as in their perception of large quantities (the counting range; [23,30,31]. **(2) The symbolic task** - Where the quantity (irrelevant dimension) is in the subitizing range in both the congruent and incongruent trials, we assumed that the DD group will show a larger congruency effect (i.e., congruent vs. incongruent) compared to the control group. We expected to see this larger effect, since DDs show a deficiency in the symbolic system [37] and their perception of small quantities (i.e., subitizing) might be intact [23,24]. Hence, we assumed that the relevant "weaker" dimension (the symbol) would be more affected by the irrelevant "strong" one (the non symbolic subitizing range). In the analysis of incongruent vs. congruent, where quantity (irrelevant dimension) is in the counting range in both the congruent and incongruent trials, we expected to find the same effect in both groups. We expected to find the same effect since the DD group is considered to be deficient in the symbolic system [37] and their perception of large quantities (the counting range) is assumed to be deficient as well [23,30,31]. In addition, we assume that in this range (i.e., the counting range) the irrelevant dimension will have a smaller effect on participants' performance, in both DD and control groups, since quantities in this counting range are not processed automatically [26].

## Method

### Participants

Fifteen adults with developmental dyscalculia (2 males, 13 females; mean age = 26 years, 2 months, SD = 3 years, 2 months) and sixteen adults without developmental dyscalculia (4 males, 12 females; mean age = 25 years, 5 months, SD= 2 years, 8 months), participated in the study. Participants gave their written consent to take part in the experiment and were paid 30 NIS as compensation. The recruitment, payment and overall procedure were authorized by the Research Ethics Committee of Haifa University.

#### Categorization and assessment criteria

In order to discard learning disabilities (LD) (to be distinguished from DD), participants were categorized using the standardized diagnostic tests from the "Israeli learning function diagnosis system" for high school and higher education students. This system is a computerized set of tests and standard questionnaires developed by the National Institute for Testing and Evaluation to diagnose learning disabilities in high school and higher education students. The tests and questionnaires are nationally normalized and hence assisted our recruitment of DD and typically developing participants.

Participants underwent numerical (simple calculation, procedural knowledge calculation, and numbers line positioning tasks), reading (text), rapid naming (of numbers and letters), phonemic awareness (phoneme omission), and attention tests (a questionnaire of their childhood and adult attention ability based on the Diagnostic and Statistical Manual of Mental Disorders [DSM]).

To be categorized as having DD, participants had to meet the following two criteria: (1) Average or higher general ability, as indexed by standardized scores of at least −1 on the reading, rapid naming, phonemic awareness, and attention tests, and (2) impaired numeracy skills, as indexed by standardized scores ≤ −1.5, of either RT or accuracy on the simple calculation and procedural knowledge tests.

To be categorized as a control group, participants had to meet the following two criteria: (1) Average or higher general ability, as indexed by standardized scores of at least −1, on the reading, rapid naming, phonemic awareness, and attention tests, and (2) Average or higher general ability, as indexed by standardized scores of at least −1 of RT or accuracy on the simple calculation and procedural knowledge tests. In addition, independent t-tests were conducted upon the different test results. The two groups were significantly different in all the numerical tests except the RT of the number line positioning task (for mean test results and p values of independent t-tests see Table 1).

**Table 1:** Mean standard score in the screening tests for each group and the average standard score of the two groups

	**Text reading**		**Rapid naming**		**Phoneme omission**		**Questionnaire**				**Simple calculation**		**Number line positioning**		**Procedural knowledge**	
	Acc	RT	Letters	Numbers	Acc	RT	A	B	C	D	Acc	RT	Acc	RT	Acc	RT
DDs	.59	.16	.66	-.04	.01	.07	-.24	-.35	.37	.14	−1.36	−1.06	−1.35	.34	−1.72	−1.31
Controls	.67	.67	1.06	.82	.45	.23	.19	.16	.31	.04	0.7	.35	1.3	.27	.67	.58
Average	.63	.41	.88	.42	.25	.16	-.01	-.08	.34	.08	-.25	-.31	-.34	.3	-.44	-.3
T	-.38	−1.21	−1.63	−3.18**	−1.78	-.59	−1.16	-.95	-.01	.36	−5.9**	−4.4**	−5.89**	-.31	−7.13**	−6.1**

Note: a, Attention in adulthood; b, Attention in childhood; c, Impulsiveness and hyperactivity in adulthood; d, Impulsiveness and hyperactivity in childhood. Sig. = An independent *T* test significant.

### The experimental task

#### Materials and methods

Stimuli were Arabic numerals (numbers 1 to 9) in different visual patterns, that appeared one after the other at the center of a computer screen (see Figure 1 for an example) (see Additional file 1 for the full list of stimuli).

Stimuli were generated using custom-written software programmed in Matlab. These routines enabled the generation of new stimuli sets for each training/experimental session. In order to eliminate the possibility that RT or acc were affected by area or density, we controlled for low visual parameters such as the density and area of the number patterns. Thus the written digits were presented in changing sizes which led to a changing amount of pixels displayed on the screen for each stimulus.

In order to create the images, the resolution is 800 × 600. Each stimuli (see Figure 1) was chosen at random by the e-prime program from a large pool of options (each quantity, 1–9 dots, includes 100 different figures per quantity).

The task itself was programmed with e-prime v2 Basic. There were two different blocks in each experiment: In the **symbolic block**, participants were asked to respond vocally and say the number appearing on the screen while ignoring the quantity (how many times it appears). In the **non symbolic block**, participants were asked to vocally decide how many times the number appears and to ignore the number itself. Vocal responses were recorded through the e-prime's response box.

In addition, the actual number that the participant said was recorded as well. It was typed by the research assistant on the computer keyboard (i.e., the research assistant sat beside the participants and typed the number the participant said or 0 if no oral response was given).

Each block began with a practice block (see Table 2 for the full list of practice stimuli which were different from those in the experimental phase but included the same conditions). Feedback was given after each trial only in the practice phase. The participants continued to the actual experiment only if they accumulated 13 correct answers in the practice phase (the practice block had 24 trails; hence 13 trials are over 50% correct answers).

**Table 2:** List of stimuli in practice trials (including description of the number of repetitions, numerical distances, and the range of each type of stimulus)

**The symbol**	**The quantity**	**Number of repetitions**	**Numerical distance**	**Range of the symbol**	**Range of the quantity**	**Congruity**
1	2	2	1	Subitize	Subitize	Incongruent
5	6	2	1	Counting	Counting	Incongruent
4	5	2	1	Subitize	Counting	Incongruent
1	4	2	3	Subitize	Subitize	Incongruent
5	8	2	3	Counting	Counting	Incongruent
4	7	2	3	Subitize	Counting	Incongruent
1	1	4	0	Subitize	Subitize	Congruent
5	5	4	0	Subitize	Subitize	Congruent
4	4	4	0	Subitize	Subitize	Congruent

#### Procedure

Participants were seated about 50 cm from the computer screen. The task assigned to participants was conducted in two separate blocks, in which they were required to name either the written digit (i.e., the symbolic task) or the quantity (the number of times that the written digit appears; i.e., the non symbolic task). Participants were asked to ignore the irrelevant dimension (i.e., the quantity in the symbolic task or the written number in the non symbolic task). Each participant completed both blocks, while half the participants began with the symbolic block and half with the non symbolic block. The experiment itself included nine breaks in each block (the symbolic or the non symbolic) that ended when participants pressed a relevant key and were limited to two minutes, as well as a break of a few minutes between the two sections. The stimuli in each block were presented in random order. Participants were asked to respond as quickly and as accurately as possible. Each trial began with a fixation point (a small white filled square) which appeared for 500 ms followed by an empty black screen for 300 ms, and then the sample quantity that appeared for 400 ms and disappeared, leaving a blank gray screen for 1500 ms (with no masking). The next trial began with the fixation point. The presentation time of the stimuli (400 ms +1500 ms blank gray screen) is based on previous studies that examined subitizing and counting [21,42,52].

### Statistical analyses

#### The variables used for the different statistical analyses

In order to explore possible different comparison processes that could be involved according to the hypotheses, the median RTs of all correct responses of each participant were entered into a 3-way repeated measurements ANOVA, with group (i.e., control or DD) as the only between-subject factor, and congruity (i.e., congruent, incongruent) and stimuli range (i.e., counting, subitizing) of the non symbolic quantity as within-subject factors. Since our hypotheses are distinct and different for each task, this 3-way analysis was conducted separately for each task, the symbolic and the non symbolic. An independent-samples *t*-test was conducted within the different conditions, with the only between-subject variable being group (i.e., control or DD).

Subitizing and counting in separation was only investigated in the quantity (non symbolic) dimension and not in the written digit (symbolic) dimension (which was always analyzed as a whole range from 1 to 9), since studies have found that the different ranges (i.e., subitizing vs. counting) yield different RTs and acc rates only in the non symbolic system [18,21,27]. To the best of our knowledge, no study has found such an effect (i.e., different patterns in small vs. large written numbers) in the symbolic system.

In addition, and since there are different numbers of stimuli in the counting and in the subitizing ranges, we analyzed these ranges separately by creating 4 different variables: (1) congruent-subitizing, (e.g., the written digit 1 appears once) (2) congruent- counting, (e.g., the digit 6 appears six times) (3) incongruent-subitizing (e.g., digits 1–9 appear three times) and (4) incongruent-counting (e.g., the digits 1–9 appear six times).

Also, for a second separate analysis, we calculated the relative dispersion of errors (RDE), which is the relative numerical difference between the correct answer and the participant’s incorrect actual answer divided by the correct answer. This variable takes into account not only the distance between a correct and incorrect response, but also where along the mental number line was the response made (i.e., magnitude). Accordingly, and to learn about the RDE, a new variable was computed using only the error trials (|participant answer- actual correct answer/correct answer|). This variable was then averaged for each of the 4 new variables created (i.e., congruent-counting, congruent-subitizing, incongruent-counting, and incongruent-subitizing).

The median RDE's of each participant was entered into a 3-way repeated measurement ANOVA, with group (i.e., control or DD) as the between- subject factor, and condition (i.e., congruent-counting, congruent-subitizing, incongruent-counting, and incongruent-subitizing) as the within-subject factor. For post-hoc tests (e.g., within a group or within a condition) we used a Bonferroni correction. In each of these comparisons there are two independent variables (e.g., two groups or two conditions). Therefore, after Bonferroni correction, the alpha value was set at 0.025 for all post hoc analyses.

## Results

The average accuracy (acc) was 75.25% (SD = .95) in the non symbolic task and 98.69% (SD =.92) in the symbolic task. There was no reaction time/accuracy tradeoff in each condition as indicated by the non significant Pearson correlations between RTs and acc (for example, the correlation between RT and acc in the congruent condition of the nonsymbolic task was R = 0.204, p = .196) (see Additional file 1: Appendix 2 for mean RT and acc rates). The RT slope show a typical subitizing - counting slope, reaction time slopes were greater for counting than for subitizing trials [F (1, 29) = 12.32, p < .001]. Planned comparisons indicated that the difference in subitizing slopes was not significant [F (1, 29) = 1.08, p = .305], whereas the difference in counting slopes was significant [F (1, 29) = 113, p < .000].

### Reaction time analyses (RT)

**Figure 2:**
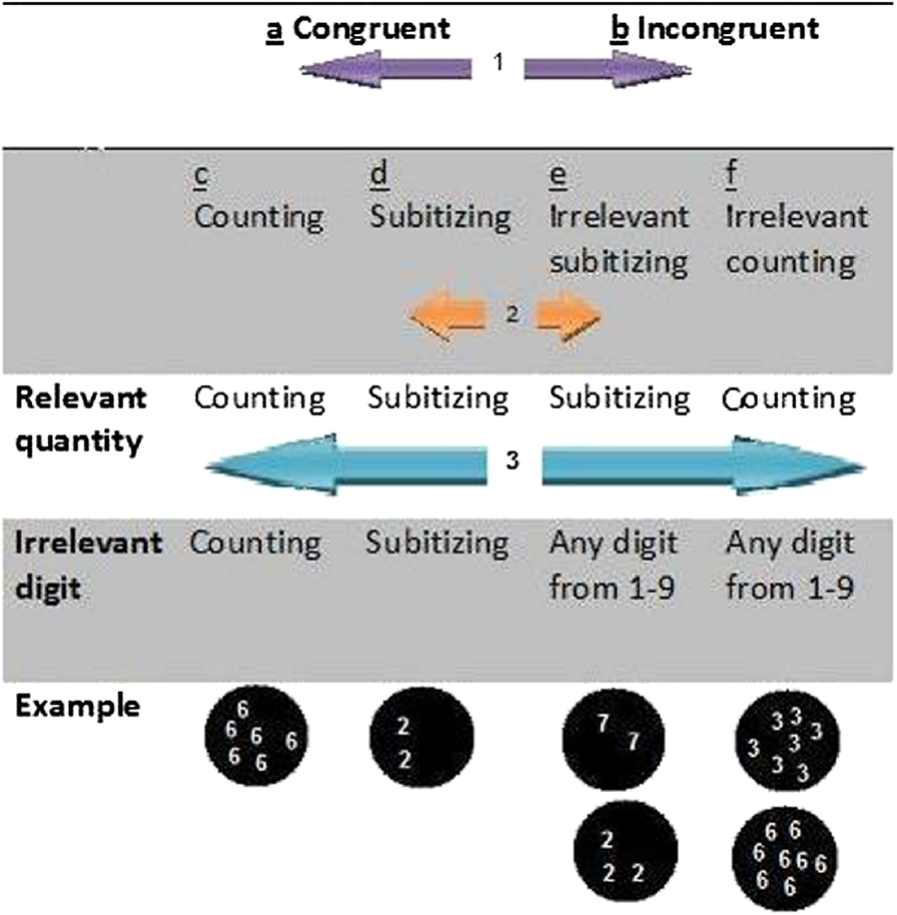
A description of the different analyses in the non symbolic task.

**Non symbolic task** (See Figure 2 for analyses and conditions)

*Analysis 1(congruent* vs. *incongruent):* The analysis indicated a marginal significant main effect of congruency [F (1, 29) = 4.122, p = .052], and no main effect of group. Since the main effect of congruency was only marginally significant, we added the likelihood ratio analysis [53]. The likelihood ratio value is larger than 1 [λc= 1.99], suggesting that the null model (e.g., the two conditions are the same) does not provide a reasonable match to the data; hence the conditions are most probably not the same.

**Figure 3:**
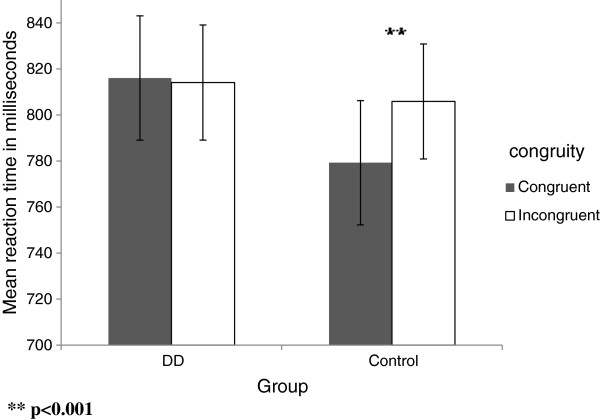
Mean RTs as a function of the congruity in the non symbolic task in DD and control group.

There was a significant interaction between group and congruity [F (1, 29) = 5.556, p = .025]. Simple effects of this interaction revealed that within the **control group** there was a significant difference between the congruent and the incongruent conditions [F (1, 15) = 9.289, p = .008], RT was shorter in the congruent condition by 26.62 ms (for mean reaction time in the task, see Additional file 1: Appendix 2a). Within the **DD group** there was no significant difference between the two conditions [p = .815], that is, RT remained the same regardless of the congruency of the stimuli (see Figure 3).

We then analyzed the interaction between group and congruity separately for each range of the relevant dimension (the quantity).

*Analysis 2 (congruent - subitizing* vs. *incongruent - subitizing):* In the **subitizing** range we compared condition d (see Figure 2), the congruent - subitizing condition (both written digit and quantity are in the subitizing range. e.g., the digit “3” appears 3 times), to condition e, the incongruent condition wherein the quantity (i.e., the relevant dimension) is in the subitizing range, regardless of the written digit (i.e., the irrelevant dimension, which could be any written digit from '1' to '9').

*Analysis 3 (congruent - counting* vs. *incongruent - counting)*. In the **counting** range we compared condition c, the congruent counting condition (both written digit and quantity are in the counting range), to condition f, the incongruent condition in which the quantity (relevant dimension) is in the counting range (any written digit from '1' to '9' appearing more than 4 times).

**Figure 4:**
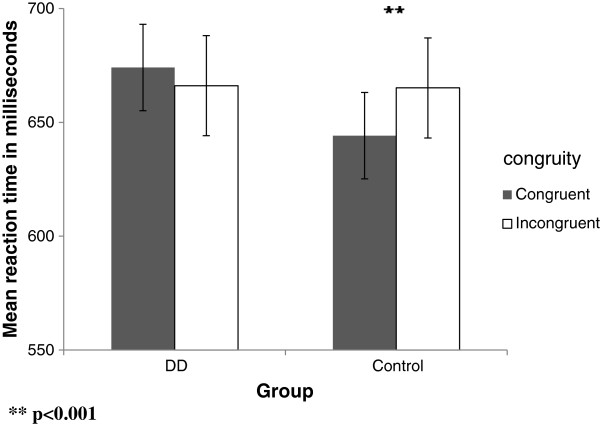
Mean RTs as a function of the congruity in the subitizing range of the non symbolic task in DD and control group.

Only in the **subitizing range** (*Analysis 2)* was the interaction between group and congruity significant [F (1, 29) = 8.366, p = .007]. We further analyzed the congruency effect in the subitizing range for each group separately and found that in the control group only, RT was significantly faster in the congruent condition [F (1, 15) = 8.657, p = .010] than in the incongruent one. There was no such significant effect in the DD group [p = .276] (see Figure 4).

**Figure 5:**
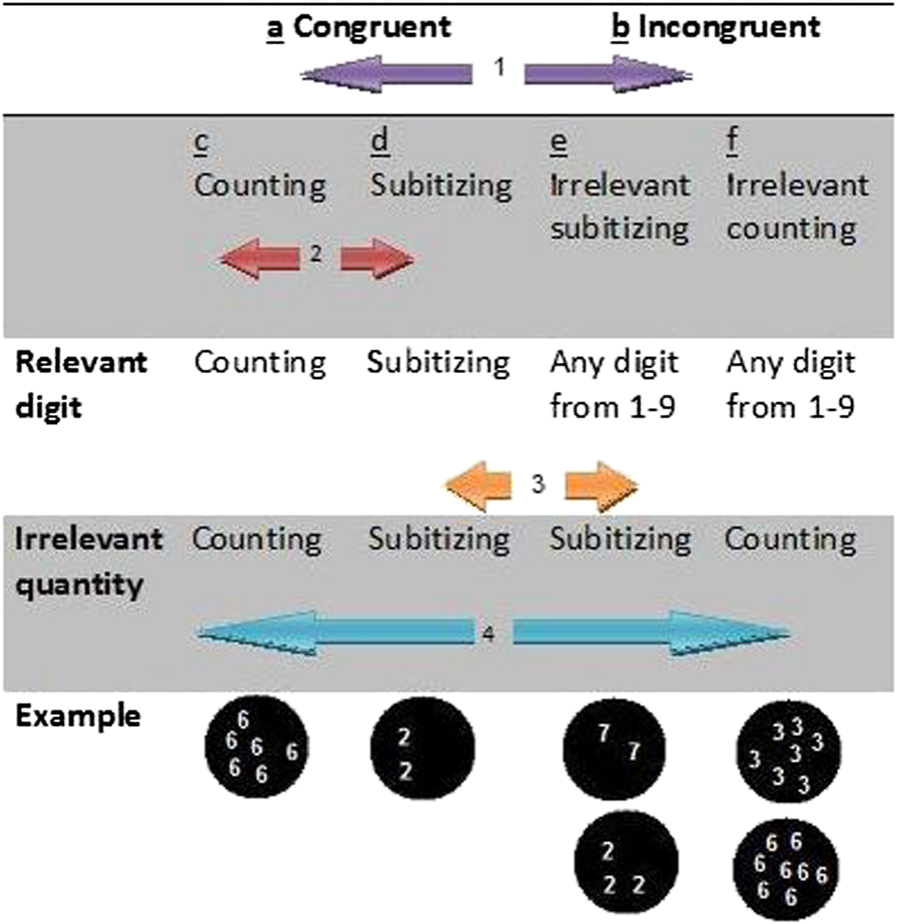
A description of the different analyses in the symbolic task.

**Symbolic task** (see Figure 5)

**Figure 6:**
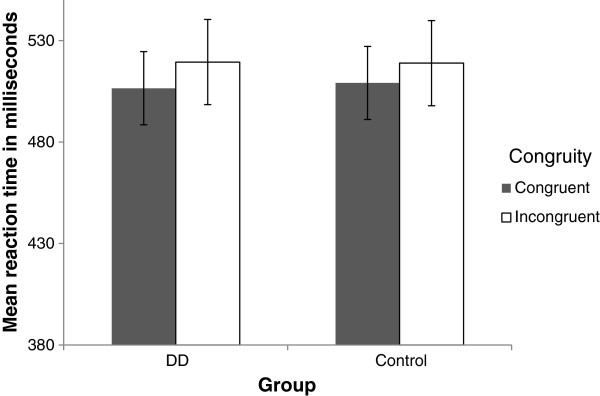
Mean RTs as a function of the congruity in the symbolic task in DD and control group.

*Analysis 1 (congruent* vs. *incongruent):* A significant main effect of congruency was found, indicating that RT was shorter in the congruent condition [F (1, 29) = 25.17, p < .001]. There was no significant interaction between group and congruity [p = .507] (see Figure 6).

*Analysis 2 (congruent - counting* vs. *congruent - subitizing)*: A main effect for range was found in the comparison of the two different ranges with **congruent** stimuli (e.g., condition c vs. condition d) [F (1, 29) = 6.608, p = .016]. Specifically, RT was shorter in the congruent subitizing range. There was no significant interaction between the two congruent conditions and group [p = .294].

Despite the fact that there was no significant interaction between group and congruency, we further analyzed each congruency effect (i.e., congruent vs. incongruent) in each group separately, due to theoretical reasons and our hypotheses.

**Figure 7:**
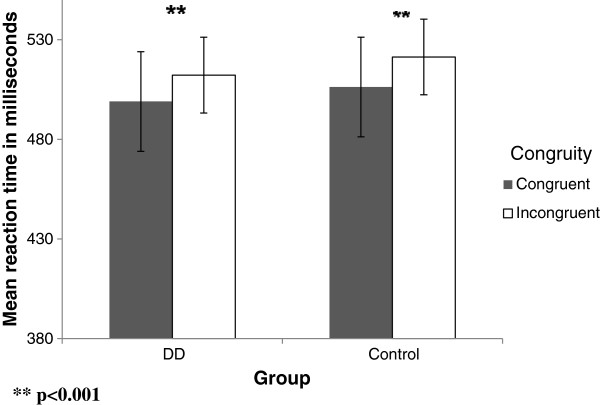
Mean RTs as a function of the congruity in the subitizing range of the symbolic task in DD and control group.

*Analysis 3 (congruent - subitizing* vs. *incongruent - subitizing)*: We compared condition d, congruent subitizing, to condition e, incongruent wherein the quantity (the irrelevant dimension) is in the **subitizing** range while the written digits (i.e., the relevant dimension) range from 1 to 9. In this comparison, there was a significant main effect [F (1, 29) = 26.158, p < .001] of congruency. Thus, when the quantity was in the subitizing range, RT was significantly shorter in the congruent condition for both groups. This was found in the control group [F (1, 15) = 23.68, p < .001] as well as in the DD group [F (1, 14) = 13.89, p = .002] (see Figure 7).

*Analysis 4 (congruent - counting* vs. *incongruent - counting)*: In contrast, when comparing condition c, the congruent counting range condition, to condition f, the incongruent where the quantity (irrelevant dimension) was in the **counting** range (for example, the relevant written digit is in the range of 1 to 9, but the irrelevant quantity dimension is in the counting range from 5–9), there was no effect, and RT was the same in the incongruent condition.

### Distance effect

A 3-way ANOVA, that included the four different distances (e.g., zero, one, two, and five) in each task range (i.e., symbolic task subitizing range, symbolic task counting range, non symbolic task subitizing range, non symbolic task counting range), was conducted in order to examine the effect of the numerical distance between the relevant and the irrelevant quantities on the performance of the two groups. Within each of the four task ranges, a repeated measurements ANOVA with group (e.g., control or DD) as the between- subject factor and distance (e.g., zero, one, two, and five) as the within-subject factor was conducted.

The performance of both groups followed the typical distance effect pattern (i.e., smaller distances are processed slower than larger ones), as indicated by the significant distance effect that appeared in each numerical range beyond group. We further analyzed these effects and found that distance five was significantly faster than a distance of two in the non symbolic task [F (1, 29) = 48.62, p < .001] and in the symbolic one [F (1, 29) = 27.04, p < .001]. Distance two was processed significantly faster than distance one in the non symbolic task [F (1, 29) = 58.36, p < .001] and in the symbolic one [F (1, 29) = 12.87, p = .001]. None of the interactions with group (e.g., control or DD) as the between- subject factor were significant, and both groups reacted in the same manner to the different distances in all the different conditions (i.e., in both of the tasks as well as in both of the ranges).

### Accuracy analyses (acc)

#### ACC - Non symbolic task

When comparing all the congruent trials with the incongruent ones, there was a main effect of congruency [F (1, 29) = 15.402, p < .001] and no interaction between group and congruency [p = .511]**.** Acc was higher in the congruent condition as compared to the incongruent condition.

#### ACC - Symbolic task

The comparison between the two congruence conditions yielded a main effect of congruency [F (1, 29) = 29, p = .042]. The interaction was not significant [p = .197].

### Relative dispersion of errors [RDE]

We analyzed the RDE [|participant answer - actual correct answer/correct answer|] of all the incorrect answers given compared to the stimuli. For each participant eight different averages (e.g., congruent, incongruent, counting range, subitizing range, congruent_counting range, congruent_subitizing range, incongruent_quantity in the counting range and incongruent_quantity in the subitizing range) were created. There was no significant difference between the two groups in their variability of responses in both the symbolic and the non symbolic tasks.

#### RDE - **Non symbolic task**

In the non symbolic task, the mean RDE of the control group was .058 (SD =.03) and for the DD group .048 (SD = .02), and the difference between the groups was not significant (p = .227). There was a significant congruency (i.e., congruent vs. incongruent) main effect [F (1, 29) =13.26, p < .001], RDE was larger in the incongruent condition. In addition, there was no significant interaction between group and congruity [p = .174]. Range (i.e., counting vs. subitizing) was significant [F (1, 29) =15.23, p = .001], with the counting condition more dispersed than the subitizing condition. There was no significant interaction between group and range either [p = .628].

When analyzing the congruity effect in the two different ranges (i.e., congruent vs. incongruent) a main effect of congruency was found in both ranges (in the subitizing range [F (1, 29) =7.67, p = .010] and in the counting range [F (1, 29) =11.04, p = .002]), with the congruent condition less dispersed than the incongruent condition. In addition, there was no significant interaction between groups and congruity in the subitizing range [p = .262] and in the counting range [p = .296].

#### RDE - Symbolic task

In the symbolic task, the mean RDE of the control group was .01 (SD = .01) and for the DD group .006 (SD = .04), where the difference between the groups was not significant (p = .207). There was a significant congruent (i.e., congruent vs. incongruent) main effect [F (1, 29) =10.59, p = .003], showing larger RDE in the incongruent condition. Additionally, there was no interaction between group and congruity [p = .398].

There was no main effect of congruency (i.e., congruent vs. incongruent) in the counting range [p = .472]. In contrast, in the **subitizing** range there was a main effect of congruency [F (1, 29) =8.29, p = .007], therefore, when the irrelevant quantity was in the subitizing range congruency lessened the RDE.

## Discussion

The present study investigated automaticity and attention in counting and subitizing ranges. The main objective was to examine whether DD adults are deficient in their ability to automatically process one or both of the numerical representation systems. The results showed a complex picture.

In general we found that, in the **non symbolic task** – the symbolic irrelevant information did influence processing of the relevant information (i.e., RT was shorter for congruent than for incongruent trials) in the control but not in the DD group. The uniqueness of this novel enumeration Stroop task enables us to look at the automaticity of counting vs. subitizing ranges separately and hence to reach a finer resolution of the automaticity of the non symbolic system. Accordingly, we found that this significant interaction between group and congruity appears mainly when the relevant dimension (quantity) was in the subitizing range (i.e., one, two, three, or four) and not when it was in the counting range (i.e., more than four). Specifically, the DD group only showed a congruency effect when the relevant non symbolic information was in the subitizing range.

**In the symbolic task,** there were no significant differences between the groups’ responses, and the two groups showed a typical congruency effect (RT was shorter for congruent than incongruent trials). In addition, when quantity, i.e. the non symbolic irrelevant dimension, was in the counting range, it didn't affect participants' performance, in both DD and control groups.

Three additional variables (besides task, congruity, and range) were examined in order to eliminate possible alternative explanations of the results: the newly computed variable (i.e., relative dispersion of errors [RDE]), accuracy rate, and the distance effect. These components appear to be a good indication of performance: RDE and accuracy changed as predicted (1) in the different tasks (more accurate and less RDE in the symbolic task), (2) in the different congruence conditions (more accurate and less RDE in the congruent condition), and (3) in the different ranges (more accurate and less RDE in the subitizing range). In addition, a typical distance effect – the smaller the distance the larger the RT - was found in the two groups. However, none of these components yielded a significant difference between the two groups, apart from one acc comparison that will be further discussed.

Notably, the current findings replicate previous ones, and hence, we consider them to be credible and all of our new findings to be highly reliable. Specifically, (1) typically, as we found, responding to congruent trials is found to be faster than responding to incongruent ones [5,42,52], (2) responses are typically, and in our findings as well, faster in the symbolic task compared to the non symbolic one [52,54], and (3) as often found previously, the subitizing range is processed faster than the counting one [19,21,27] (for mean reaction times and accuracy rates see Additional file 1: Appendix 2). (4) The RT slope for both groups show a typical logarithmic subitizing - counting slope, indicating that participants use their subitizing and counting abilities [20,21,42].

We will now discuss three main components of the task which may have influenced performance and current results, namely, dimension (i.e., symbolic vs. non symbolic), congruency (i.e., congruent vs. incongruent), and numerical ranges (i.e., subitizing vs. counting).

### Non symbolic task

In the **non symbolic** task, only the control group showed a congruency effect, while the DD group did not. The novel enumeration Stroop tasks let us not only examine the degree of automaticity of the two systems but also examine the different ranges of these systems (e.g., subitizing vs. counting) more specifically. In contrast to the control group, the DD group was influenced by the irrelevant symbolic dimension only when the quantity (relevant dimension) was in the subitizing range (i.e., 1–4) (DDs showed a congruency effect in the counting range only).

To date, and to the best of our knowledge, no study has tested the automaticity of symbolic and non symbolic representations in DDs separately for the subitizing and the counting ranges. This may be one reason why different scientific findings are not coherent and conclusive regarding the core deficit of DDs; while some studies support a deficit in the symbolic system as the main deficit [37,38] others argue that it is in the non symbolic system [12,21,55].

It is also notable that the lack of a congruency effect is all the more unexpected when taking into consideration that the non symbolic task required more time than the symbolic one for both groups (that is, reaction times were significantly shorter in the symbolic than the non symbolic task; see Additional file 1: Appendix 2a, c;). This means that written digits might be processed faster than quantities in the DD group, and yet written digits (the symbolic irrelevant information) did not influence the outcome of the non symbolic task. That is to say, the DD group did not process the symbolic information automatically.

Two possible explanations for a non significant congruency effect in the subitizing range in DDs are plausible. First, people with DD may not perceive written digits as automatically as the control group. Therefore, written digits have a smaller amount of influence on the "stronger" non symbolic dimension, quantity in the subitizing range. Indeed, the quantity dimension may be considered a strong one, since there is much evidence that subitizing is a fast, resilient [18,21,27], and automatic process [19]. Previous studies support the hypothesis that DDs are not deficient in their abilities to subitize [23,31,32]. Studies have also shown that automatic access to symbolic numbers is deficient in dyscalculic adults [12,21,55], and that DD children suffer mainly from deficient representations in the symbolic system [37,38,56].

Secondly, it is possible that the representation of the symbolic system is intact, but the ability to associate between the symbolic (e.g., the symbol “3”) and the non symbolic systems (e.g., the quantity of 3 items) is deficient in dyscalculia. Consequently, even if symbolic processing is automatic and intact, the weak association between quantity and symbols is not sufficiently strong, and hence, the irrelevant symbolic dimension does not interfere with or facilitate the subitizing range. Several studies support this weak association theory. To begin with, the association between these two systems is accepted [35,36]. In addition, some researchers, based on their findings, have suggested that the association between the symbolic and the non symbolic system is low in DD participants [12,25]. Much like the first explanation, this weaker association is not strong enough to influence the "stronger" non symbolic dimension, quantity in the subitizing range.

When the relevant dimension was in the counting range (i.e., more than 4) there was no interaction between group and congruency. Namely, both groups showed a congruency effect, suggesting that the irrelevant symbolic information automatically influenced the relevant non symbolic information in the counting range, in both groups. Studies have shown that the ability to count may be deficient in DDs [23,30-32], and hence, even though the symbolic system (or the association between the symbolic and the non symbolic systems, as suggested above) may be deficient, it is still processed in a way that influences the low and deficient counting process. That is, in the counting range, the two dimensions of the stimuli (both the symbolic system and the non symbolic) are deficient, which might lead to a pattern of results similar to that of the control group (for illustration see Additional file 1: Appendix 2c).

### Symbolic task

No interaction was found between group and congruity in the **symbolic** task. That is, both groups were similarly influenced by the irrelevant non symbolic dimension. Specifically, a main effect of congruity (shorter RT in the congruent compared to the incongruent condition) was found for both groups when the quantity was in the subitizing range and not when it was in the counting range.

These findings are in contradiction with our assumption. We assumed that we would find a reversed effect to that seen in the non symbolic task. If the symbolic system of the DD group is indeed deficient [37,38], as suggested above, then in the symbolic task the DD group should have been more easily influenced by the irrelevant non symbolic dimension (quantity) than the control group. The expected stronger congruency effect was even more probable in the subitizing range of the irrelevant non symbolic dimension (the quantity) given that this range is found to be intact in DDs [23,30-32]. There is a likelihood, as previously proposed, that it is not the symbolic system itself that is deficient rather the ability to associate between the two systems. Neuroimaging studies provide us with a few examples of asymmetric associations between the symbolic and the non symbolic systems [57,58]. For instance, Piazza et al. [56] used fMRI adaptation of a group of dots and of Arabic digits; they found that an adaptation for a group of dots (i.e., non symbolic dimension) led to adaptation of digits, but this effect was not found in the other direction. The findings of the current study can be a result of such asymmetric association between quantity and its representative symbol. This association may be deficient in the DD group when a written digit is presented and the meaning or the representative quantity needs to be retrieved, but not in the other direction, that is, when a quantity is presented and the written digit needs to be retrieved.

Furthermore, none of the groups were affected by the irrelevant non symbolic dimension (i.e., quantity) when it was in the counting range. That is, large quantities are not automatically interpreted as exact numbers, and therefore do not influence the processing of the relevant symbolic information. Similar patterns of results were found in previous studies as well [26,27]. Notice that this pattern too is not necessarily bidirectional; that is, large quantities are linked to an exact written digit in the non symbolic task, as can be seen from the congruency effect found for both groups in the quantity range in the non symbolic task (i.e., when it is relevant). This suggests that symbolic information was automatically processed and interfered with the processing of large non symbolic quantities.

It should also be noted that the symbolic task is processed faster than the non symbolic one, in both groups (see Additional file 1: Appendix 2a, c). Nonetheless, and despite this fact, the process of written digits (the symbolic irrelevant information) was influenced by the irrelevant quantity (in the non symbolic task). These results are compatible with previous studies, which showed an automatic process for both the symbolic and the non symbolic number systems in adults [12,21,42,55]. However, automaticity does not always follow an "order rule", meaning that the faster processed feature is not necessarily the one that will influence the slower processed one. For example, Henik and Tzelgov [42] found, much like the current results, a surprising interference of the irrelevant slower process on the relevant faster one. They interpreted this finding as an indication of parallel processing of symbolic and non symbolic information, rather than serial processing. The current findings support this parallel processing, since in the symbolic task the slower irrelevant non symbolic dimension influenced the faster relevant symbolic one in both groups. In addition, in the non symbolic task a faster irrelevant symbolic dimension didn't influence the slower processing of the relevant non symbolic one in the DD group.

### Distance effect

In this study four different distances were used; congruent - zero which appears only in the congruent trials (e.g., the number “9” appears nine times), and three incongruent distances that obviously appear only in the incongruent trials: one, two, and five. Notably, contrary to other types of Stroop task, in the current enumerations Stroop task participants are not asked to compare numbers but to name the written digit or the quantity. Accordingly, the distance in the current task is between the relevant and the irrelevant dimension of the same stimulus (as opposed to a typical and more familiar numerical distance between two numbers compared in a typical comparison task [59]), which are both different in their number format and part of different systems (symbolic or nonsymbolic). Hence, this is a complex and different example of distance effect.

Some studies have argued that DDs display stronger distance effect compared to control groups [3,5,30]. In order to further study this assumption, and to examine whether a qualitative difference between DDs and controls may be the cause of the results, we looked at the different distances of the task (zero, one, two, and five) and compared them without referring to the congruency of the stimuli. This was done separately for each range and for the two ranges combined. A similar pattern of the distance effect was found for both groups (the larger the distance, the shorter the RT) and no interaction was found between the different distances and groups. Hence, the different numerical distances between the relevant and irrelevant dimensions have no effect on our results and were not the reason for the differences between the groups. That is, the distance effect is not the cause for the differences, rather they are caused by the inability to automatically process numerical symbolic information and non symbolic large quantities. These findings are in line with previous studies which examined distance effect in DD participants and did not find a different pattern of distance effect as compared to control groups [39].

## Conclusions

**Figure 8:**
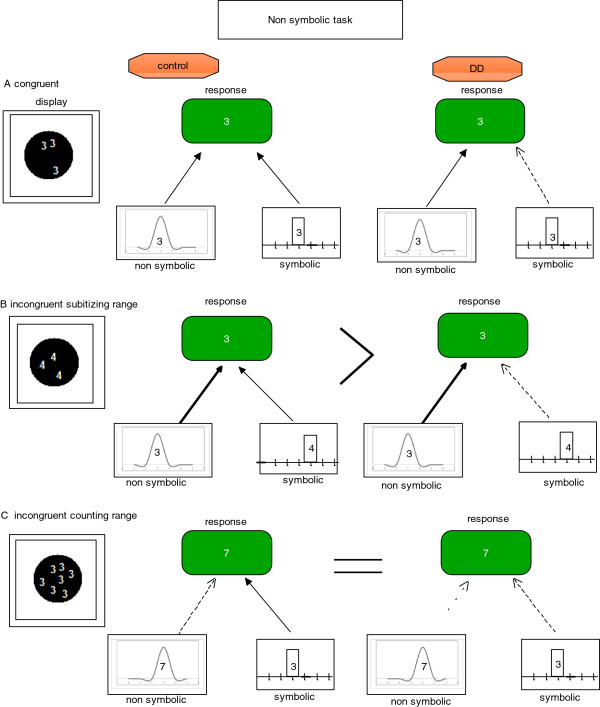
A tentative model of the results.

In summary, the results show a distinct difference in the non symbolic task of the enumeration Stroop between DD and control adults. As opposed to previous studies, the current task enables us to carefully study not only the symbolic and non symbolic numerical systems in general but also the different numerical ranges. The current findings clearly suggest that the symbolic system of the DD group is deficient and also that the counting range of the non symbolic system is deficient as well. We formed a theoretical model of these results in the non symbolic task, which may act as a framework for future scientific work. The model illustrates that the symbolic system of the DD group may be deficient, but the non-symbolic subitizing range is intact. The model also illustrates that counting abilities may be deficient in both symbolic and non-symbolic numerical representations (see Figure 8).

We have also proposed that this deficiency is not necessarily absolute, and hence DDs are not deficient in their perception of the symbolic system per se but rather in their association between a written digit and its corresponding quantity. This deficiency is not necessarily bidirectional, therefore it is possible that the association between a written digit and its corresponding quantity is weak while the association between a quantity and its corresponding written digit is intact. Finally, in the symbolic task both groups (i.e., controls and DDs) showed a congruency effect (i.e., RT was shorter in the congruent than in the incongruent condition).

## Competing interests

We declare that we have no competing interests.

## Authors' contributions

The work presented here was carried out in collaboration between all authors. TF and OR defined the research theme, designed methods and experiments, analyzed the data, interpreted the results and wrote the paper. TF carried out the laboratory experiments. Both authors have contributed to, seen and approved the manuscript."

## Supplementary Material

Additional file 1 Appendix 1. Click here for file

## References

[B1] Butterworth B Campbell JID Developmental dyscalculia Handbook of mathematical cognition 2005 New York: Psychology Press 455 467

[B2] Rotzer S Kucian K Martin E Von Aster M Klaver Loenneker T Optimized voxel-based morphometry in children with developmental dyscalculia Neuroimage 2008 39 417 422 10.1016/j.neuroimage.2007.08.045 17928237

[B3] Price G Holloway I Rasanen P Vesterinen M Ansari D Impaired parietal magnitude processing in developmental dyscalculia Curr Biol 2007 17 24 R1042 R1043 10.1016/j.cub.2007.10.013 18088583

[B4] Kaufmann L Wood G Rubinsten O Henik A Meta-analyses of developmental fMRI studies investigating typical and atypical trajectories of number processing and calculation Dev Neuropsychol 2011 36 763 787 10.1080/87565641.2010.549884 21761997

[B5] Stroop JR Studies of interference in serial verbal reactions J Exp Psychol 1935 18 643 662

[B6] Dehaene S Dehaene-Lambertz G Cohen L Abstract representations of numbers in the animal and human brain Trend Neurosci 1998 21 355 361 10.1016/S0166-2236(98)01263-6 9720604

[B7] Gallistel CR Gelman R Holyoak K, Morrison R Mathematical cognition The Cambridge handbook of thinking and reasoning 2005 New York: Cambridge University Press 559 588 23

[B8] Dehaene S The number sense how the mind creates mathematics 1997 New York: Oxford University Press 13 63

[B9] Brannon EM Abbott S Lutz DJ Number bias for the discrimination of large visual sets in infancy Cognition 2004 93 B59 B68 10.1016/j.cognition.2004.01.004 15147939

[B10] Lipton J Spelke E Origins of number sense: large number discrimination in human infants Psychol Sci 2003 14 396 401 10.1111/1467-9280.01453 12930467

[B11] Lipton J Spelke E Discrimination of large and small numerosities by human infants Infancy 2004 5 271 290 10.1207/s15327078in0503_2

[B12] Rubinsten O Henik A Automatic activation of internal magnitudes: a study of developmental dyscalculia Neuropsychology 2005 19 5 641 648 16187882 10.1037/0894-4105.19.5.641

[B13] Rubinsten O Henik A Double dissociation of functions in developmental dyslexia and dyscalculia J Educat Psychol 2006 98 854 867

[B14] Kucian K Loenneker T Martin E Aster M Non symbolic numerical distance effect in children with and without developmental dyscalculia: a parametric fMRI study Dev Neuropsychol 2011 36 6 741 762 10.1080/87565641.2010.549867 21761996

[B15] Piazza M Facoetti A Trussardi AN Berteletti I Conte S Lucangeli D Dehaene S Zorzi M Developmental trajectory of number acuity reveals a severe impairment in developmental dyscalculia Cognition 2010 116 33 41 10.1016/j.cognition.2010.03.012 20381023

[B16] Mazzocco MM Feigenson L Halberda J Impaired acuity of the approximate number system underlies mathematical learning disability (dyscalculia) Child Dev 2011 82 1224 1237 10.1111/j.1467-8624.2011.01608.x 21679173 PMC4411632

[B17] Kucian K Loenneker T Dietrich T Dosch M Martin E Aster M Impaired neural networks for approximate calculation in dyscalculic children: a functional MRI study Behav Brain Fun 2006 2 31 48 10.1186/1744-9081-2-31 PMC1574332 16953876

[B18] Mandler G Shebo BJ Subitizing: an analysis of its component processes J Exp Psychol: General 1982 111 1 22 10.1037//0096-3445.111.1.1 6460833

[B19] Kaufman EL Lord MW Reese TW Volkmann J The discrimination of visual number Amer J Psychol 1949 62 498 525 10.2307/1418556 15392567

[B20] Trick LM Pylyshyn ZW What enumeration studies can show us about spatial attention: evidence for limited capacity pre attentive processes J Exp Psychol Hum Percept Perform 1993 19 2 331 351 8473843 10.1037//0096-1523.19.2.331

[B21] Koontz KL Berch DB Identifying simple numerical stimuli: processing inefficiencies exhibited by arithmetic learning disabled children Math Cogn 1996 2 1 24 10.1080/135467996387525

[B22] Schleifer P Landerl K Subitizing and counting in typical and atypical development Dev Sci 2011 14 280 291 22213901 10.1111/j.1467-7687.2010.00976.x

[B23] Desoete A Gregoire J Numerical competence in young children and in children with mathematics learning disabilities Learn Indiv Differ 2006 16 351 367 10.1016/j.lindif.2006.12.006

[B24] Fischer B Gebhardt C Hartnegg K Subitizing and visual counting in children with problems in acquiring basic arithmetic skills Optom Vis Dev 2008 39 1 24 29

[B25] Wilson AJ Revkin SK Cohen D Cohen L Dehaene S An open trial assessment of “the number race”, an adaptive computer game for remediation of dyscalculia Behav Brain Fun 2006 2 1 20 10.1186/1744-9081-2-20 PMC1523349 16734906

[B26] Logie RH Baddeley AD Cognitive processes in counting J Exp Psychol 1987 13 2 310 326

[B27] Trick LM Pylyshyn ZW Why are small and large numbers enumerated differently? a limited capacity preattentive stage in vision Psychol Rev 1994 101 80 102 8121961 10.1037/0033-295x.101.1.80

[B28] Revkin SK Piazza M Izard V Cohen L Dehaene S Does subitizing reflect numerical estimation? Psychol Sci 2008 19 607 614 10.1111/j.1467-9280.2008.02130.x 18578852

[B29] Hyde DC Spelke ES All numbers are not equal: an electrophysiological investigation of small and large number representations J Cogn Neurosci 2009 21 1039 1053 10.1162/jocn.2009.21090 18752403 PMC2735795

[B30] Geary DC Mathematics and learning disabilities J Learn Disabil 2004 37 4 15 10.1177/00222194040370010201 15493463

[B31] Dowker A Individual differences in arithmetic: implications for psychology, neuroscience and education 2005 Hove, East Sussex: Psychological Press

[B32] Geary DC Bow-Thomas CC Yao Y Counting knowledge and skill in cognitive addition: a comparison of normal and mathematically disabled children J Exp Child Psychol 1992 54 372 391 10.1016/0022-0965(92)90026-3 1453139

[B33] Dehaene S Spelke E Pinel P Stanescu R Tsivkin S Sources of mathematical thinking: behavioral and brain-imaging evidence Science 1999 284 970 974 10.1126/science.284.5416.970 10320379

[B34] Hurford JR Language and nmber: The emergence of a cognitive system 1987 Oxford: Basil Blackwell Press

[B35] Piazza M Pinel P Le Bihan D Dehaene S A magnitude code common to numerosities and number symbols in human intraparietal cortex Neuron 2007 53 293 305 10.1016/j.neuron.2006.11.022 17224409

[B36] Notebaert K Nelis S Reynvoet B The magnitude representation of small and large symbolic numbers in the left and right hemisphere: an event-related fMRI study J Cogn Neurosci 2011 23 3 622 630 10.1162/jocn.2010.21445 20201630

[B37] Rousselle L Noel MP Basic numerical skills in children with mathematics learning disabilities: a comparison of symbolic vs. nonsymbolic number magnitude processing Cognition 2007 102 3 361 395 10.1016/j.cognition.2006.01.005 16488405

[B38] Mussolin C Martin R Schiltz C Relationships between number and space processing in adults with and without dyscalculia Acta Psychol 2011 138 1 193 203 10.1016/j.actpsy.2011.06.004 21802651

[B39] Landerl K Kolle C Typical and atypical development of basic numerical skills in elementary school J Exp Child Psychol 2009 103 4 546 565 10.1016/j.jecp.2008.12.006 19254797

[B40] Mussolin S Mejias Noel MP Symbolic and nonsymbolic number comparison in children with and without dyscalculia Cognition 2010 115 1 10 25 10.1016/j.cognition.2009.10.006 20149355

[B41] Noel MP Rousselle L Developmental changes in the profiles of dyscalculia: An explanation based on a double exact-and-approximate number representation model Front Hum Neurosci 2011 5 165 22203797 10.3389/fnhum.2011.00165 PMC3243900

[B42] Henik A Tzelgov J Is 3 greater than 5 – the relation between physical and semantic size in comparison tasks Mem Cognit 1982 10 389 395 10.3758/BF03202431 7132716

[B43] Szucs D Soltesz F Event-related potentials dissociate facilitation and interference effects in the numerical Stroop paradigm Neuropsychologia 2007 45 3190 3202 10.1016/j.neuropsychologia.2007.06.013 17675108

[B44] Soltesz F Szucs D Dekany J Markus A Csepe V A combined event-related potential and neuropsychological investigation of developmental dyscalculia Neurosci Lett 2007 417 181 186 10.1016/j.neulet.2007.02.067 17367929

[B45] Holloway ID Ansari D Developmental specialization in the right intraparietal sulcus for the abstract representation of numerical magnitude J Cogn Neurosci 2009 22 11 2627 2637 19929327 10.1162/jocn.2009.21399

[B46] Girelli L Lucangeli D Butterworth B The development of automaticity in accessing number magnitude J Exp Child Psychol 2000 76 104 122 10.1006/jecp.2000.2564 10788305

[B47] Rubinsten O Henik A Berger A Shahar-Shalev S The development of internal representations of magnitude and their association with Arabic numerals J Exp Child Psychol 2002 81 74 92 10.1006/jecp.2001.2645 11741375

[B48] Mussolin C De Volder A Grandin C Schlogel X Nassogne MC Noel MP Neural correlates of symbolic number comparison in developmental dyscalculia J Cogn Neurosci 2010 22 860 874 10.1162/jocn.2009.21237 19366284

[B49] Bachot J Gevers W Fias W Roeyers H Number sense in children with visuospatial disabilities: orientation of the mental number line Psychol Sci 2005 47 172 183

[B50] Pavese A Umiltà C Symbolic distance between numerosity and identity modulates stroop interference J Exp Psychol Hum Percept Perform 1998 24 1535 1545 9988600 10.1037//0096-1523.24.5.1535

[B51] Pavese A Umiltà C Further evidence on the effects of symbolic distance on Stroop-like interference Psychol Res 1999 62 62 71 10.1007/s004260050040

[B52] Naparstek S Henik A Count me in! On the automaticity of numerosity processing J Exp Psychol Learn Mem Cogn 2010 36 4 1053 1059 20565221 10.1037/a0019766

[B53] Glover S Dixon P Likelihood ratios: a simple and flexible statistic for empirical psychologists Psychon Bull Rev 2004 11 791 807 10.3758/BF03196706 15732688

[B54] Holloway ID Ansari D Mapping numerical magnitudes onto symbols: the numerical distance effect and individual differences in children’s mathematics achievement J Exp Child Psychol 2008 103 1 17 29 18513738 10.1016/j.jecp.2008.04.001

[B55] Butterworth B The mathematical brain 1999 London: Macmillan press

[B56] Kaufmann L Vogel S Starke M Kremser C Schocke M Wood G Developmental dyscalculia: compensatory mechanisms in left intraparietal regions in response to nonsymbolic magnitudes Behav Brain Fun 2009 5 35 10.1186/1744-9081-5-35 PMC2731029 19653919

[B57] Eger E Michel V Thirion B Amadon A Dehaene S Kleinschmidt A Deciphering cortical number coding from human brain activity patterns Curr Biol 2009 19 1608 1615 10.1016/j.cub.2009.08.047 19781939

[B58] Verguts T Fias W Representation of number in animals and humans: a neural model J Cogn Neurosci 2004 16 1493 1504 10.1162/0898929042568497 15601514

[B59] Moyer RS Landauer TK The time required for judgements of numerical inequality Nature 1967 215 1519 1520 10.1038/2151519a0 6052760

