# Supplementary material for: Symbolic and non symbolic numerical representation in adults with and without developmental dyscalculia
Source: Behav Brain Funct. 2012 Nov 28;8:55. doi: 10.1186/1744-9081-8-55 (PMC3527185; doi:10.1186/1744-9081-8-55)
Supplement: Additional file 1 — Appendix 1. [file 1744-9081-8-55-S1.docx]

**Appendix 1**

List of the stimuli in the experiment (including number of repetitions, numerical distance and the range of each stimulus).

| The symbol | The quantity | Number of repetition | Numerical distance | Range of the symbol | Range of the quantity | Congruity |
| --- | --- | --- | --- | --- | --- | --- |
| 1 | 2 | 12 | 1 | Subitize | Subitize | Incongruent |
| 2 | 1 | 12 | 1 | Subitize | Subitize | Incongruent |
| 3 | 4 | 12 | 1 | Subitize | Subitize | Incongruent |
| 4 | 3 | 12 | 1 | Subitize | Subitize | Incongruent |
| 6 | 7 | 12 | 1 | Counting | Counting | Incongruent |
| 7 | 6 | 12 | 1 | Counting | Counting | Incongruent |
| 8 | 9 | 12 | 1 | Counting | Counting | Incongruent |
| 9 | 8 | 12 | 1 | Counting | Counting | Incongruent |
| 1 | 3 | 12 | 2 | Subitize | Subitize | Incongruent |
| 3 | 1 | 12 | 2 | Subitize | Subitize | Incongruent |
| 2 | 4 | 12 | 2 | Subitize | Subitize | Incongruent |
| 4 | 2 | 12 | 2 | Subitize | Subitize | Incongruent |
| 6 | 8 | 12 | 2 | Counting | Counting | Incongruent |
| 8 | 6 | 12 | 2 | Counting | Counting | Incongruent |
| 7 | 9 | 12 | 2 | Counting | Counting | Incongruent |
| 8 | 7 | 12 | 2 | Counting | Counting | Incongruent |
| 1 | 6 | 12 | 5 | Subitize | Counting | Incongruent |
| 6 | 1 | 12 | 5 | Counting | Subitize | Incongruent |
| 2 | 7 | 12 | 5 | Subitize | Counting | Incongruent |
| 7 | 2 | 12 | 5 | Counting | Subitize | Incongruent |
| 3 | 8 | 12 | 5 | Subitize | Counting | Incongruent |
| 8 | 3 | 12 | 5 | Counting | Subitize | Incongruent |
| 4 | 9 | 12 | 5 | Subitize | Counting | Incongruent |
| 9 | 4 | 12 | 5 | Subitize | Subitize | Incongruent |
| 1 | 1 | 12 | 0 | Subitize | Subitize | Congruent |
| 2 | 2 | 36 | 0 | Subitize | Subitize | Congruent |
| 3 | 3 | 36 | 0 | Subitize | Subitize | Congruent |
| 4 | 4 | 36 | 0 | Subitize | Subitize | Congruent |
| 6 | 6 | 36 | 0 | Counting | Counting | Congruent |
| 7 | 7 | 36 | 0 | Counting | Counting | Congruent |
| 8 | 8 | 36 | 0 | Counting | Counting | Congruent |
| 9 | 9 | 36 | 0 | Counting | Counting | Congruent |

**Appendix 2.** Means of median RTs and accuracy rates as a function of Task congruity and range in DD and control groups.

| Incongruent-digit-  subitizing | Incongruent-digit-counting | Incongruent-quantity-  subitizing | Incongruent-quantity-counting | Congruent-subitizing | Congruent-counting | Incongruent | Congruent |  |
| --- | --- | --- | --- | --- | --- | --- | --- | --- |
|  |  | a. Non symbolic task – mean RT (ms) | | | | | | |
| 893.46  (129.31) | 893.46  (98.73) | 666.09 (112.02) | 1156.40  (170.12) | 674.33  (105.52) | 1059.22  (112.69) | 814.07  (114.32) | 816.07 (149.01) | DD |
| 863.24  (137.51) | 665.72  (180.09) | 665.72  (85.55) | 1087.92  (221.17) | 643.77  (91.21) | 990.39  (172.28) | 805.89  (112.05) | 779.27  (155.87) | Control |
|  |  | b. Non symbolic task – mean acc (%) | | | | | | |
| 71.11  (5.31) | 68.22  (12.03) | 96.86  (4.04) | 75.12  (1.04) | 98.01  (2.13) | 51.65  (18.01) | 70.08  (8.72) | 77.01  (11.32) | DD |
| 74.05  (8.75) | 74.13  (4.49) | 97.94  (7.43) | 75.04  (3.01) | 97.32  (3.21) | 61.12  (15.34) | 75.14  (9.74) | 79.34  (16.21) | Control |
|  |  | c. Symbolic task – mean RT (ms) | | | | | | |
| 509.80  (79.64) | 523.01  (93.45) | 526.95  (126.09) | 508.46  (134.51) | 499.06  (98.21) | 512.91  (113.12) | 507.77  (115.92) | 506.51 (105.12) | DD |
| 523.79  (110.91) | 515.82  (84.93) | 531.34  (70.01) | 505.58  (56.08) | 506.30  (54.64) | 512.02  (66.13) | 518.87  (63.12) | 509.03  (60.12) | Control |
|  |  | d. Symbolic task – mean acc (%) | | | | | | |
| 99.10  (0.98) | 98.31  (1.88) | 98.92  (2.42) | 99.12  (1.01) | 99.05  (1.20) | 99.25  (1.31) | 98.87  (2.12) | 99.17  (1.00) | DD |
| 98.49  (1.24) | 98.01  (1.31) | 98.51  (2.05) | 98.71  (2.11) | 98.01  (1.03) | 98.83  (2.41) | 98.43  (2.45) | 98.37  (1.92) | Control |

Note: Standard deviations are shown in parentheses
